# Emotion recognition from body movement through interpretable motion-aware sequential modeling

**DOI:** 10.3389/frai.2026.1897208

**Published:** 2026-08-06

**Authors:** Sergio Esteban-Romero, Iván Martín-Fernández, Rubén San-Segundo, Manuel Gil-Martín, Fernando Fernández-Martínez

**Affiliations:** Grupo de Tecnología del Habla y Aprendizaje Automático (THAU Group), Information Processing and Telecommunications Center, E.T.S.I. de Telecomunicación, Universidad Politécnica de Madrid (UPM), Madrid, Spain

**Keywords:** affective computing, body motion, emotion recognition, multiple instance learning, transformers

## Abstract

Emotion recognition from bodily movement remains a challenging problem, particularly when only pose-based motion sequences are available and emotionally informative content is not uniformly distributed across time. In this work, we propose a Window Transformer architecture grounded in the Multiple Instance Learning (MIL) paradigm to address this challenge. Rather than processing the full sequence as a single temporal stream, the model decomposes it into overlapping windows and learns to assign greater relevance to those segments containing stronger emotional content. This formulation provides a more interpretable framework, since the learned relevance scores reveal which temporal regions drive the final prediction, while also yielding richer, context-aware representations of each segment. We evaluate the proposed approach on two publicly available datasets, MEED and DIEM-A, and compare it against a standalone Transformer baseline under different batch size and window configuration settings. The Window Transformer consistently outperforms the baseline and exhibits a more stable behavior across training configurations, achieving best accuracies of 56.35 ± 2.67% on MEED and 23.84 ± 0.83% on DIEM-A in a subject-independent scenario. Beyond performance gains, this work also establishes new benchmark values on both datasets, providing reference results that future research on bodily emotion recognition can build on.

## 1 Introduction

Emotions play a critical role in the way humans interact with their environment being fundamental for interpersonal relationships (Lu et al., 2025b; Chittepu et al., 2026). The recent surge of highly capable conversational agents has further pushed the boundaries of Human-Computer Interaction (HCI), increasing the demands for systems that can effectively adapt to the emotional states of the user in order to support more natural and socially aware interactions (Lithoxoidou et al., 2025). Providing such systems with emotional capabilities could be especially beneficial in domains such as healthcare, education (Bouhlal et al., 2020), and psychological diagnosis and therapy, where sensitivity to affective signals is often essential (Inkster et al., 2018; Striegl et al., 2024).

Human emotions, however, are expressed through multiple modalities, including audio, through prosodic and paralinguistic information, visual signals, such as facial expressions and body movements, and linguistic content, conveyed by semantic and syntactic patterns (Luna-Jiménez et al., 2021; Esteban-Romero et al., 2024; Wu et al., 2025). Recent advances in Deep Learning (DL) have substantially improved the recognition capabilities of affective systems by enabling the joint processing of these heterogeneous sources of information, leading to increasingly powerful multimodal models with strong representational capacity (Han et al., 2025; Xu et al., 2025). Nevertheless, the availability and suitability of each modality may vary considerably depending on the application context, especially in scenarios where privacy or trustworthiness constraints are critical.

In the context of rapidly evolving multimodal conversational agents, such as ChatGPT (OpenAI, 2024) and Gemini (Gemini, 2025), most recent advances have focused on textual, visual, and audio modalities to address broad real-world interaction needs. In particular, Vision-Language Models (VLMs) and related multimodal architectures have demonstrated a remarkable ability to learn rich transferable representations and to support natural downstream interactions (Assran et al., 2025; Qwen, 2025). However, these gains are often accompanied by a substantial increase in the number of parameters and, consequently, in the computational cost and opacity of the resulting systems (Xiao et al., 2025). This motivates the design of lightweight architectures that remain computationally feasible while preserving recognition performance. In the specific domain of body-movement analysis, recent work has also begun to adapt LLMs to motion understanding: Wang R. et al. (2026) use a VLM to caption RGB frames and project the resulting features, together with body-landmark features, into the latent space of a Qwen3-4B model for Human Action Recognition (HAR), illustrating the growing interest in combining language-model reasoning with skeletal motion representations.

Despite the progress achieved in multimodal emotion recognition, body movement remains a particularly relevant but still underexplored source of affective information. Unlike static visual cues, bodily emotions are expressed through dynamic changes in posture, gesture intensity, movement amplitude, body orientation, and temporal coordination (Poyo Solanas et al., 2020; Zhang et al., 2023). However, these emotional cues are not necessarily present across the entire motion sequence, but may emerge only during specific expressive moments. This observation motivates the use of Multiple Instance Learning (MIL), where the sequence is treated as a set of temporal segments and the model learns which of them are more relevant for the final decision (Carbonneau et al., 2018; Del Gaudio et al., 2026). In addition, pose and motion-based representations offer a promising alternative to raw RGB video in privacy-sensitive domains, since they preserve relevant behavioral information while reducing the exposure of identifiable visual details (Moon et al., 2023; Gao et al., 2025). Conveniently, the MIL formulation also provides a natural source of interpretability, since the relevance scores assigned to each temporal segment reveal which parts of the sequence drive the final prediction.

At the same time, these challenges also highlight the importance of interpretability and explainability. In sensitive domains such as HCI and healthcare, it is often not sufficient for a model to simply output a right prediction, it is also necessary to understand which parts of the observed behavior contributed to that decision and whether the reasoning process is aligned with meaningful human-interpretable cues (Abdelhalim et al., 2026). This need becomes even more urgent as modern multimodal architectures grow in complexity, reinforcing the importance of designing emotion recognition systems that are not only accurate, but also privacy-aware, computationally feasible, and interpretable. To this end, we validate our approach on two datasets that cover complementary motion acquisition paradigms: the Multi-view Emotional Expressions Dataset (MEED) (Zhang et al., 2023), where body landmarks are extracted from RGB video via pose estimation, and the Diverse Intercultural E-Motion Database of Asian Performers (DIEM-A) (Cheng et al., 2025), where motion is recorded directly using wearable motion-capture sensors, ensuring that the findings are not tied to a single data acquisition setup.

The main contributions of this work are as follows:

- Lightweight Transformer architectures for bodily emotion recognition from motion sequences extracted from actor performances.
- The problem is formulated in a weakly supervised MIL paradigm, allowing the model to estimate the relevance of different temporal regions of the sequences and their contribution to the final emotion prediction.
- An interpretable framework that captures the temporal evolution of emotional content throughout the sequence.
- The approach is validated on MEED and DIEM-A, establishing benchmark values on both datasets that future research on bodily emotion recognition can build on.

The remainder of this work is organized as follows. Section 2 reviews the related literature that supports our analysis. Section 3 describes the architecture of the proposed MIL-based model. Section 4 describes the evaluated datasets, reports the performance of the system in the considered environments and provides an analysis of the obtained results. Finally, Section 5 summarizes the main findings of this study.

## 2 Related work

Most efforts in advancing emotion recognition are built upon visual and audio modalities due to the high influence emotions have in body aspects such as facial expressions or variation in tone or prosody in the case of speech. Such relation has been widely explored in neuroscience and further supported with the surge of a large number of datasets for tasks such as Speech Emotion Recognition (SER) or Multimodal Emotion Recognition (MER) (Tripathi et al., 2018; Livingstone and Russo, 2018; Lehnen et al., 2025). However, the interaction between motor behavior and emotional processing is not yet adequately characterized, despite increasing scientific attention to its role in affective expression and perception (Sowden et al., 2021; Adlou et al., 2025).

### 2.1 Motion acquisition and body-based emotion recognition

Traditionally, motion capture data is acquired either via computer vision algorithms applied to RGB video or through physical wearable sensors. Vision-based approaches leverage deep learning architectures like MediaPipe (Lugaresi et al., 2019), ViTPose Xu et al. (2022), or OpenPose Cao et al. (2019) to extract skeletal coordinates. While non-intrusive, these models suffer from occlusions, extreme viewing angles, or rapid movements, which introduce tracking noise that can distort downstream temporal motion descriptors like velocity and acceleration. Conversely, wearable solutions, ranging from inertial measurement units to near-infrared tracking devices (Gil-Martín et al., 2024; Esteban-Romero et al., 2025), bypass visual occlusions but introduce physical setup constraints.

Regardless of the extraction paradigm, both modalities ultimately yield sequence-based landmark positions sampled over time. This common temporal structure historically motivated the deployment of Recurrent Neural Networks (RNNs) and Long Short-Term Memory (LSTM) networks to capture dynamic movement patterns (Bhatia et al., 2022; Wu et al., 2025). More recently, Transformer architectures with self-attention mechanisms have replaced RNNs, offering superior capacity for modeling long-range spatial and temporal dependencies in bodily expressions (Paiva et al., 2023). The H2OFormer architecture explores the relationships between body joints through hypergraphs, a generalization of conventional graphs, for micro-gesture emotion recognition within a hybrid-supervised framework (Xia et al., 2026). The model combines a self-supervised autoencoder, which learns robust representations for micro-gesture analysis, with a supervised classification head that uses the resulting latent representations for emotion recognition. In this way, the architecture is trained by jointly optimizing reconstruction and classification losses, encouraging the model to preserve subtle movement details during reconstruction while also learning discriminative features for the recognition task.

To augment these models, recent strategies have explored pairing sequence features with Large Language Models (LLMs) to enhance interpretation and contextual framing (Lu et al., 2025a). Recent approaches, such as the Explainable Affective Body Expression Recognition (EABER) framework, introduced a Multi-Scale Convolutional Mamba Network (MSCMNet) in combination with a Large Language Model that is adapted via Low-Rank adaptation (Hu et al., 2021) to generate textual descriptions of the motion sequences (Wang T. et al., 2026), achieving state-of-the-art performance in multiple bodily emotion recognition benchmarks. However, this explainability component relies on a total of 450 manually annotated skeleton-text pairs across the evaluated datasets. This reflects a broader limitation in the field, as bodily emotion datasets rarely provide fine-grained annotations linking motion patterns to semantic or affective descriptions. Consequently, developing and validating interpretable models remains challenging. Moreover, LLM-based approaches introduce additional computational overhead and require careful motion-text alignment to reduce hallucination, leaving room for more efficient sequence-modeling alternatives (Lu et al., 2025a).

### 2.2 Multiple instance learning for temporal and affective data

Long temporal sequences also introduce an additional challenge, namely that emotional information is not necessarily expressed uniformly throughout the entire sample. In many cases, only a subset of temporal regions contains the motion patterns that are truly informative for the underlying affective state, while other segments may correspond to neutral transitions, low-expressivity intervals, or contextual movement with limited emotional relevance (Luo et al., 2020). This makes the problem naturally compatible with the Multiple Instance Learning (MIL) paradigm, where a sequence can be interpreted as a bag of temporal instances and the model learns which segments contribute most to the final decision.

In the Supervised Fine-Tuning (SFT) paradigm, a single instance is directly associated with a particular label. However, this assumption may be suboptimal in scenarios where the most representative class features correspond to local patterns rather than to the whole input. In such cases, the lack of manually annotated labels at the instance level makes the MIL paradigm a suitable alternative, as it allows the model to learn from coarse sample-level supervision while identifying which internal regions contribute most to the final prediction (Quellec et al., 2017; Waqas et al., 2024). Under this formulation, a sample can be represented as a bag of smaller instances, and the objective is to determine which of them contain the most relevant information for the task.

Such methodology has been successfully applied to both images and videos. In medical imaging, for example, MIL has been used to detect local regions containing potential anomalies without requiring manual annotation at the patch level (Quellec et al., 2017). In sequential settings, a similar intuition has been explored in the weakly supervised temporal action localization literature, where long videos are decomposed into temporal segments and the model learns which of them are most relevant for classification and localization despite relying only on sequence-level labels (Jin et al., 2025; Zhang and Yao, 2026; Yun et al., 2024).

This perspective is particularly appealing for affective computing, since emotional information is not always expressed uniformly throughout the whole sequence, but may instead be concentrated in specific temporal regions. In this sense, MIL-based formulations not only provide a natural way to model local emotional saliency in sequential data such as speech or body keypoints, but also introduce a useful degree of interpretability by highlighting which temporal segments are driving the prediction (Mao et al., 2020). Although recent affective-computing work has begun to explore similar ideas in facial video and fine-grained weakly supervised emotion recognition (Jin et al., 2025; Del Gaudio et al., 2026), its application to bodily emotion recognition from motion sequences remains still limited, which motivates the formulation proposed in this work.

### 2.3 Privacy-aware, pose-based approaches

When emotion recognition is addressed from video, most existing approaches rely on directly processing RGB signals. For instance, a facial emotion recognition framework based on RGB and thermal image fusion achieved 95% classification accuracy on the KDEF dataset (Lundqvist et al., 1998), showing that directly processing visual appearance can be effective but also dependent on identifiable facial cues (Tran et al., 2026). Recent RGB-based approaches such as MAE-DFER (Sun et al., 2023) exploit facial video frames through masked autoencoder-style Transformer learning to capture appearance and temporal facial motion cues for dynamic facial emotion recognition.

While effective, this choice may not be optimal in domains such as healthcare or psychological assessment, where individuals may feel uncomfortable or self-conscious when being continuously recorded by cameras (Ferreira et al., 2025). In such settings, pose estimation offers an appealing alternative, as it enables the transformation of raw visual input into abstract body representations that preserve relevant movement patterns while reducing the exposure of identifiable visual details (Gao et al., 2025). This makes body-based emotion recognition particularly attractive for privacy-sensitive applications, where the goal is to infer emotional or behavioral states while minimizing intrusiveness and preserving user trust (Marrah et al., 2026).

### 2.4 Summary of prior work

Overall, previous work supports the relevance of body movement as an affective signal and highlights the potential of sequential models for emotion recognition from motion data. However, most approaches still process the sequence as a whole, without explicitly considering that emotional information may be concentrated in specific temporal regions. In this context, MIL-based temporal relevance estimation remains limited in bodily emotion recognition. This motivates the Window Transformer proposed in this work, which combines temporal windowing and relevance estimation to improve recognition while providing a more interpretable view of the motion segments involved in the final prediction.

## 3 Model architecture

**Figure 1:**
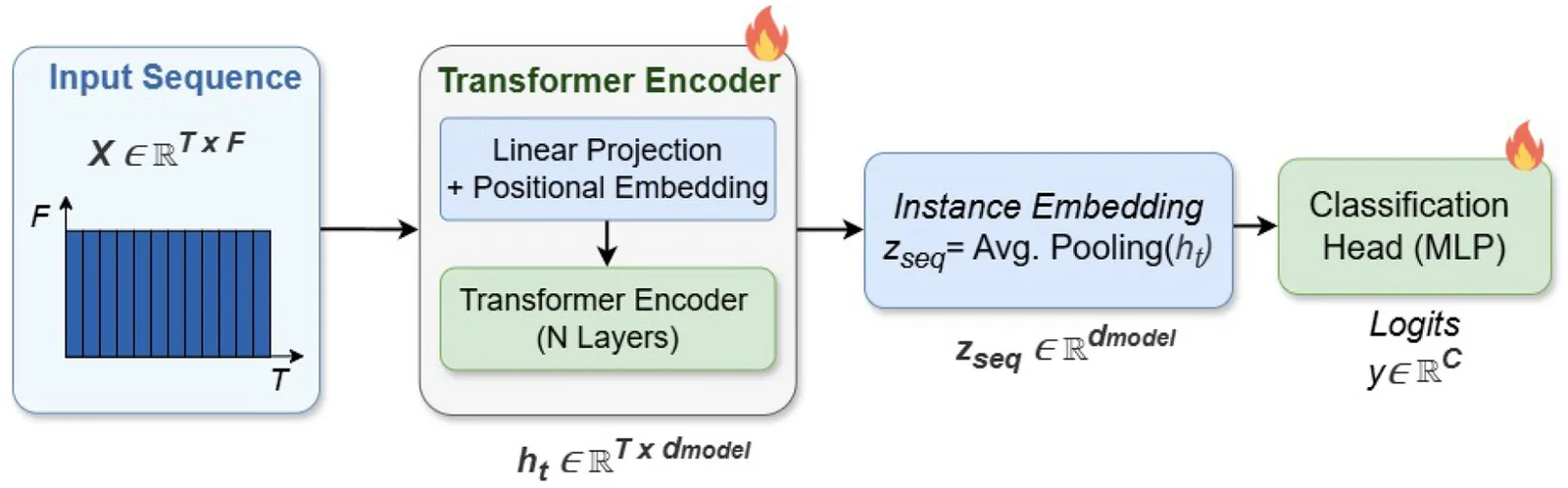
Overview of the baseline architecture. A temporal sequence X is processed by a Transformer and projected into a *d*_*model*_-dimensional space. *T* denotes the number of frames and *F* denotes the input feature dimension per frame. The sequence of hidden states, denoted as *h*_*t*_, is aggregated via mean Pooling which assigns the same relevance to every element in the sequence. The resulting Instance embedding, *z*_*seq*_, is used for final classification into *C* classes.

As a baseline, we consider a Transformer encoder architecture (Vaswani et al., 2017) that directly processes the full sequence, and the resulting hidden states are aggregated through a mean pooling layer, followed by a MLP classification head. This baseline will allow a fair comparison with the proposed MIL-based aggregation while still taking advantage of the temporal relationship patterns learned by the attention heads of the Transformer encoder. The baseline architecture is represented in Figure 1.

**Figure 2:**
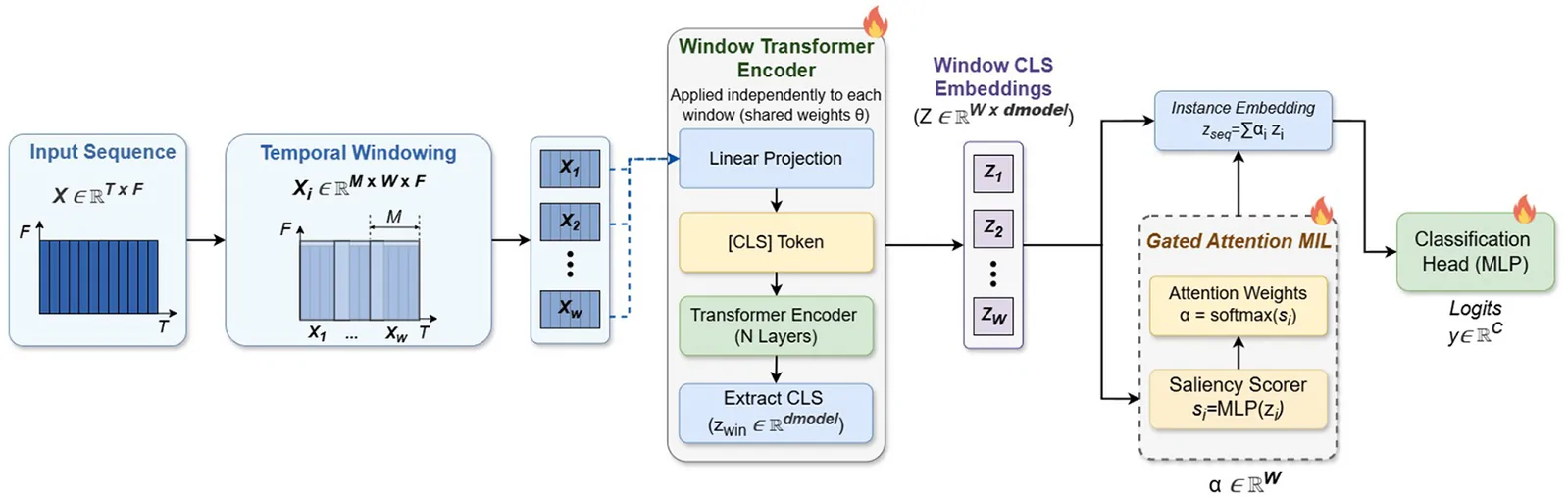
Overview of the proposed architecture. A temporal sequence *X* is unfolded into *W* windows of length *M* and projected into a *d*_*model*_-dimensional space. *T* denotes the number of frames and *F* denotes the input feature dimension per frame. A Transformer encoder leverages a [*CLS*] token to extract instance embeddings *z*_*i*_ for each window. A Gated Attention MIL head assigns saliency scores α to these instances, producing an attention-weighted sequence representation *z*_*seq*_ for final classification into *C* classes.

The proposed Window Transformer is designed around the observation that emotionally informative content is not uniformly distributed across a motion sequence (Carbonneau et al., 2018). Rather than processing the full sequence as a single temporal stream, the model decomposes it into *W* overlapping windows of length *M*, enabling it to focus on the segments that are most relevant for the final prediction, as illustrated in Figure 2. A more detailed description of the encoder and how it processes an input sequence is given in Section 3.1. The Gated Attention MIL module, which learns to assign greater importance to specific segments of the input sequence, is described in Section 3.2.

As input, each frame is represented by a flattened feature vector constructed from the landmark positions, together with their first- and second-order temporal derivatives, namely velocity and acceleration. In this way, the model receives not only the spatial configuration of the body but also explicit information about how that configuration evolves over time. The construction of this descriptor is detailed in Section 3.3.

### 3.1 Window transformer encoder

**Figure 3:**
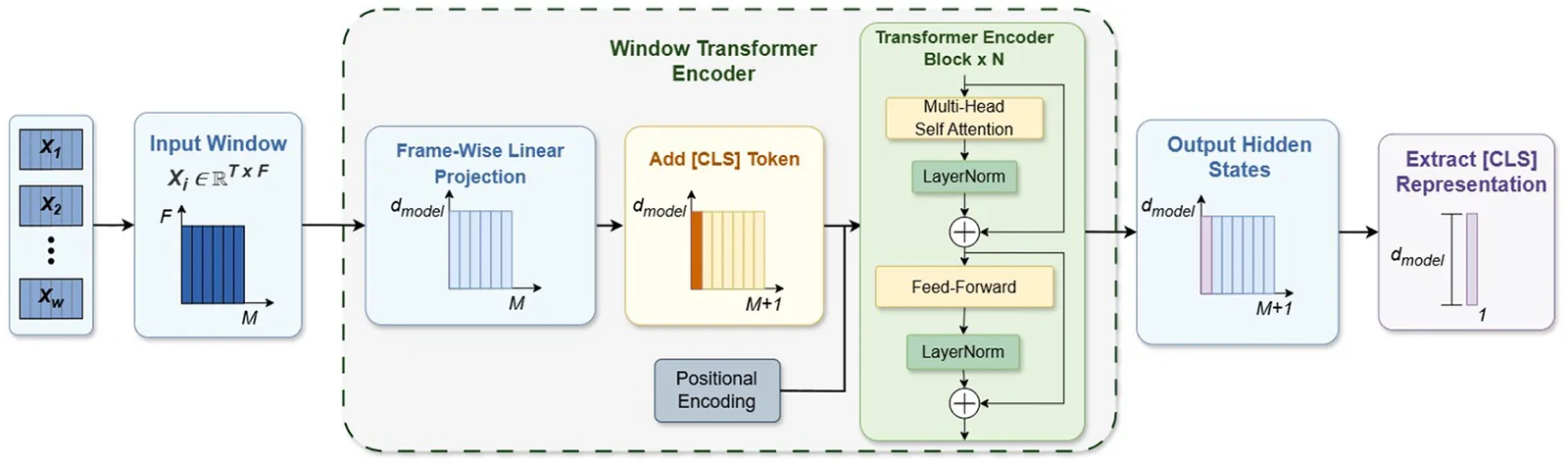
Detailed architecture of the Window Transformer Encoder. Each temporal window is processed independently using shared encoder weights. The input window is first projected into the model space and enriched with positional information. A learnable [CLS] token is then prepended to the sequence and processed through a stack of Transformer encoder layers. The final [CLS] representation is extracted as the window-level embedding used by the subsequent MIL module.

The detailed architecture of the Window Transformer Encoder is illustrated in Figure 3. The key idea of the encoder is that, given an original sequence of keypoints *X* divided into *W* windows of length *M*, the model processes the *W* windows independently. For instance, if the sequence is divided into *W* = 4 windows, then four window-level representations will be obtained.

Given a set of windows *X*_*i*_, with *i* ∈ {1, …, *W*}, each window *X*_*i*_ is first projected into the internal model dimensionality *d*_model_. Then, a learnable token, named [*CLS*], following the nomenclature of state-of-the-art architectures such as BERT and Vision Transformers (ViT) (Devlin et al., 2019; Dosovitskiy et al., 2020), is prepended to the projected sequence. Positional encoding is subsequently added to the resulting sequence, so that both the temporal tokens and the [*CLS*] token are processed within the same positional representation space.

The resulting sequence is processed by the Transformer Encoder Block, which models the temporal dependencies among the time instants within each window, rather than across the full sequence. From this final representation, the processed [*CLS*] token is extracted and used as the compact representation of the corresponding window. Therefore, the number of [*CLS*] representations matches the number of windows obtained at the beginning of the process. The relevance of these window-level representations with respect to the complete motion sequence is subsequently learned by the Gated Attention MIL module.

### 3.2 Gated attention MIL

MIL is a weakly supervised learning framework commonly applied when the training data are organized into sets, known as bags, and labels are only available at the bag level, while the individual instances contained in each bag remain unlabeled (Carbonneau et al., 2018). In our case, each complete motion sequence is interpreted as a bag, since the emotion label is associated with the whole sequence. The temporal windows extracted from that sequence are then treated as instances within the bag. Therefore, although the model knows the final emotion category of the complete motion sequence, it does not have explicit annotations indicating which specific windows contain the most relevant emotional information.

The process through which the representation for each window is obtained is described in Section 3.1. Given a set of windows *X*_*i*_, with *i* ∈ {1, …, *W*}, the Window Transformer Encoder outputs a set of window-level [*CLS*] embeddings, denoted as **Z** = {*z*_1_, …, *z*_*W*_}, where each *z*_*i*_ captures the temporal relationships within the range of its corresponding window.

Instead of performing a simple mean pooling operation over the resulting window representations, which would assign equal importance to all windows in the sequence, a Gated Attention MIL module is introduced following the MIL formulation. The objective of this module is to assign greater relevance to the most representative windows, namely those that contain the most informative time instants for predicting the final class associated with the entire motion sequence. For example, in a motion sequence labeled as happiness, windows containing neutral or transitional movements may receive lower attention weights, whereas windows showing more expressive movements, such as celebratory gestures or pronounced body activation, may be assigned higher relevance by the model.

**Figure 4:**
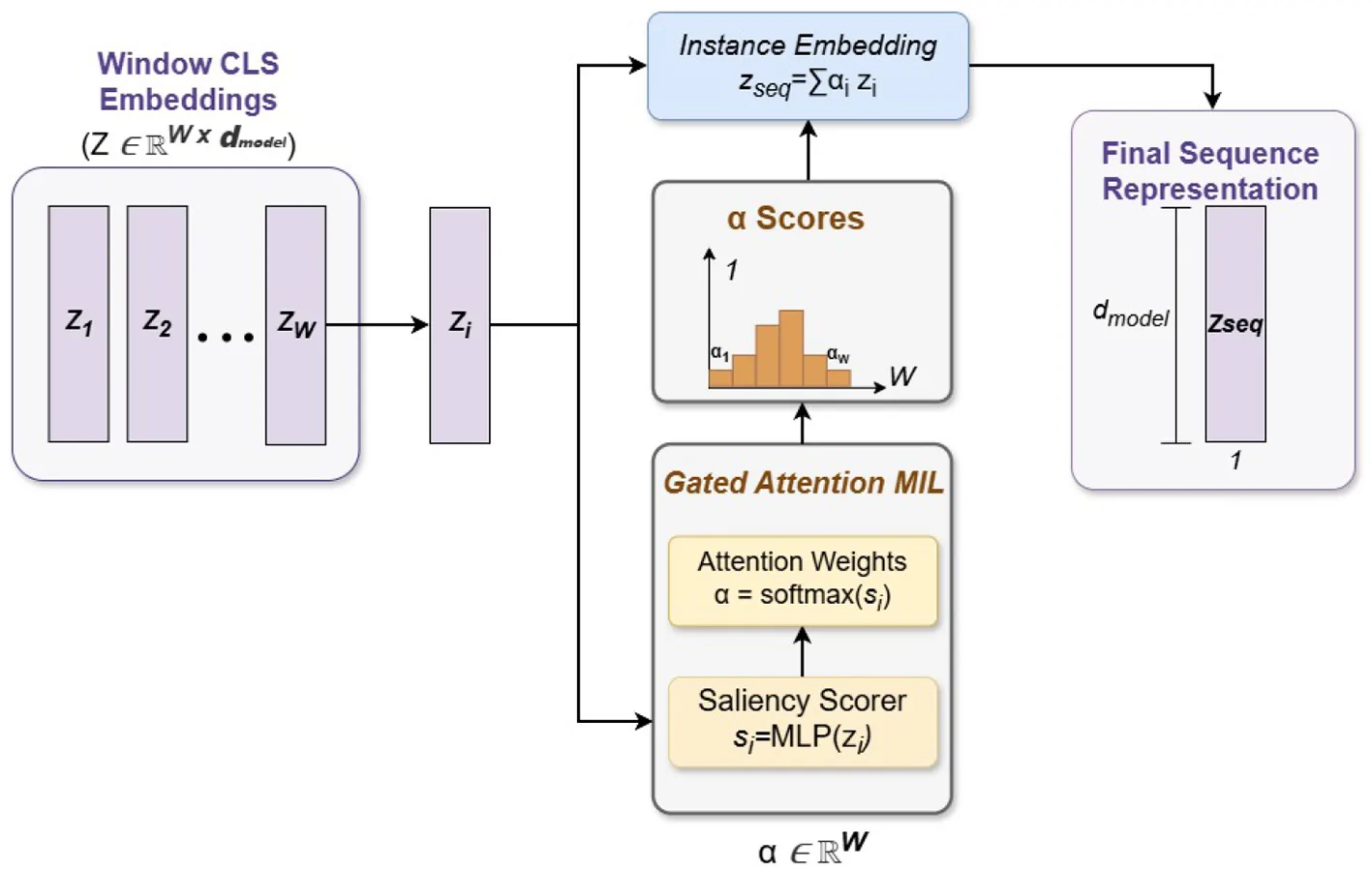
Detailed architecture of the Gated Attention MIL module. The set of window-level [CLS] embeddings is treated as a bag of temporal instances. For each instance, a gated attention mechanism estimates its individual relevance. The resulting attention weights (α) are used to obtain a weighted sequence representation, that integrates the weighted information of each window, which is finally passed to the classification head for emotion recognition.

The Gated Attention MIL module, depicted in Figure 4, processes each window-level [*CLS*] representation *z*_*i*_ through a learnable attention mechanism. This mechanism produces a relevance score for each window, which is then normalized through a softmax operator to obtain the attention weights α_*i*_, with *i* ∈ {1, …, *W*}. Each attention weight α_*i*_ is then used to perform a weighted pooling operation over the window-level [*CLS*] embeddings:


$$z_{\text{seq}}=\overset{W}{\underset{i=1}{\sum }}\alpha_{i}z_{i}.$$

Consequently, the resulting sequence-level representation *z*_seq_ integrates information from all the initially defined windows, aggregated according to their relevance for predicting the class associated with the whole motion sequence.

### 3.3 Motion descriptor construction

Let a motion position sequence be composed of *T* frames and *L* landmarks. The position of landmark *l* at time step *t* is represented as


$$\begin{array}{l} {\text{p}}_{t}^{\left(l\right)}={\left[x_{t}^{\left(l\right)},y_{t}^{\left(l\right)},z_{t}^{\left(l\right)}\right]}^{⊤}\in {\mathbb{R}}^{d},\;d\in \left\{2,3\right\}, \end{array}$$ (1)

where *d* = 2 in MEED, as landmarks are extracted from 2D video, and *d* = 3 in DIEM-A, where depth is also captured by the motion-capture sensors.

To enrich the representation with motion information, we compute first and second order temporal derivatives for each landmark independently. The velocity of landmark *l* at time step *t* is estimated using a first-order finite difference:


$$\begin{array}{l} {\text{v}}_{t}^{\left(l\right)}={\text{p}}_{t}^{\left(l\right)}-{\text{p}}_{t-1}^{\left(l\right)},\;t=2,\ldots ,T. \end{array}$$ (2)

Likewise, the acceleration is computed as the finite difference of consecutive velocities:


$$\begin{array}{l} {\text{a}}_{t}^{\left(l\right)}={\text{v}}_{t}^{\left(l\right)}-{\text{v}}_{t-1}^{\left(l\right)}={\text{p}}_{t}^{\left(l\right)}-2{\text{p}}_{t-1}^{\left(l\right)}+{\text{p}}_{t-2}^{\left(l\right)},\;t=3,\ldots ,T. \end{array}$$ (3)

The input descriptor associated with frame *t* is then formed by concatenating, for all landmarks, the position, velocity, and acceleration vectors:


$$\begin{array}{l} {\text{x}}_{t}=\text{conca}{\text{t}}_{l=1}^{L}\left({\text{p}}_{t}^{\left(l\right)},{\text{v}}_{t}^{\left(l\right)},{\text{a}}_{t}^{\left(l\right)}\right). \end{array}$$ (4)

This produces a flattened frame-level representation in which both spatial configuration and temporal evolution are explicitly encoded using position, velocity, and acceleration information.

Since velocity and acceleration are undefined for the first one and two frames respectively, the concatenation in Equation 4 is only defined from *t* = 3 onward, yielding a sequence of effective length *T*′ = *T*−2. Zero-padding the initial frames would be an alternative, but truncation is preferred to avoid introducing artificial motion descriptors at sequence onset.


$$\begin{array}{l} X={\left\{{\text{x}}_{t}\right\}}_{t=3}^{T}, \end{array}$$ (5)

### 3.4 Model parameters

Table 1 shows that all four configurations are of a similar order of magnitude, ranging from approximately 1.12M to 1.26M trainable parameters. The main source of variation comes from the input representation, since DIEM-A uses 57 landmarks while MEED uses only 25, which increases the size of the input projection and related components. Nevertheless, despite this difference in input dimensionality, the models remain broadly comparable in terms of overall parameter count.

**Table 1:** Comparison of trainable parameters by module across model configurations.

Model	Encoder	Sal. scorer	Classifier	Others	Total
MEED (transformer only)	1 M	0	34 K	38 K	1.12 M
DIEM-A (transformer only)	1 M	0	34 K	131 K	1.22 M
MEED (with saliency scorer)	1 M	33 K	34 K	43 K	1.16 M
DIEM-A (with saliency scorer)	1 M	33 K	34 K	140 K	1.26 M

For transformer-only models, the saliency scorer is absent and therefore set to 0. The “Others” category includes class tokens, positional embeddings, input projection layers, and pooling layers when applicable.

## 4 Results

In this section, we first introduce the two datasets used in this study, which provide complementary settings for evaluating motion analysis and affective behavior under different acquisition conditions. We then describe the experimental protocol, including the training configuration and evaluation metrics employed throughout the study. Finally, we present and discuss the results obtained with the proposed models, with particular attention to their performance on the MEED and DIEM-A datasets.

### 4.1 Datasets

We evaluate the proposed approach on two publicly available datasets that differ in their motion acquisition paradigm. MEED provides 2D landmark sequences extracted from RGB video via pose estimation, while DIEM-A provides 3D landmark sequences recorded directly using wearable motion-capture sensors. This difference in acquisition modality makes the two datasets complementary, allowing us to assess the generality of the proposed approach across distinct data collection setups.

#### 4.1.1 Multi-view emotional expressions dataset

The MEED dataset (Zhang et al., 2023) comprises 4, 102 multi-view recordings (front, left, and right views) representing seven emotion classes: six different emotional body movements (Anger, Disgust, Fear, Happiness, Sadness, and Surprise) and Neutral. Specifically, the actors wear black tights and perform for six seconds according to a randomly presented scenario, among 5 different scenarios defined for each emotion, or a free performance for a given emotion, but always facing the frontal camera. The scenarios cover daily event situations, such as being in a quarrel and threatening to fight back for anger, pursuing an attacker in the case of fear, or encountering an old friend not seen in years and being very pleased to see them, which represents happiness (De Gelder and Van den Stock, 2011). The recordings contain the frames extracted by OpenPose (Cao et al., 2019) and the coordinates in the 2D pixel space for the 25 body joints.

From the dataset description, we observe that some files were lost due to malfunctioning of the recording equipment resulting in 1, 386, 1, 326, and 1, 390 videos for left, front, and right views, respectively. Consequently, we define the experimentation based on the frontal view to mitigate the impact of missing videos as it is the view with fewer examples. An additional advantage of basing the analysis on the frontal view is that we are also reducing the effect of missing keypoints, which is significantly lower than in the side views. Hence, the actor “F04” has been removed from the experiments, as it does not contain frontal view examples. The resulting dataset contains examples of 21 different subjects.

**Figure 5:**
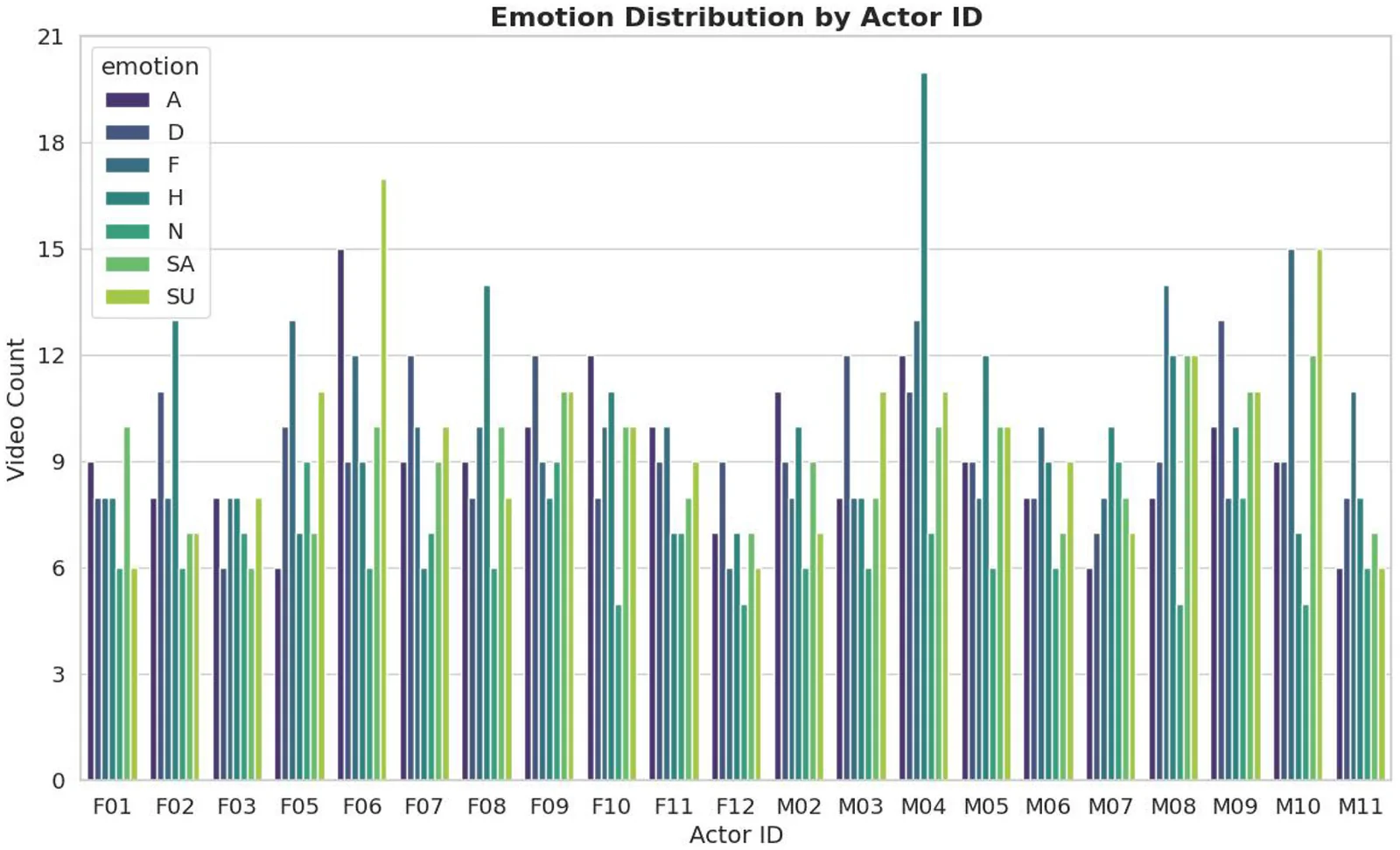
Distribution of videos for each emotion grouped by users in the MEED dataset using only “Front” view.

The imbalance in the number of emotions performed by each user is another concern arising from missing videos, as observed in Figure 5, where the distribution of videos for each emotion and for each user in the MEED dataset is represented. There are specific users, such as “M04” with considerably more “Happy (H)” videos, or “F06” with more “Surprise (SU)” examples. From the 1, 326 videos selected for our experiments, 1, 324 are standardized to 97 frames, while two videos contain 87 and 75 frames each.

#### 4.1.2 Diverse intercultural e-motion database of Asian performers

The DIEM-A database (Cheng et al., 2025) contains high-quality motion-capture data from 97 Asian professional performers (54 subjects from Japan, 43 subjects from Taiwan). The performers were requested to perform 3 self-created scenarios for 12 different emotions at 3 different intensity levels, except for “Neutral,” resulting in 10, 767 motion recordings. The dataset covers 13 emotion classes: the 7 basic emotions (Joy, Sadness, Anger, Surprise, Fear, Disgust, and Contempt) defined by Ekman and Friesen (Ekman and Friesen, 1986), 5 social emotions (Gratitude, Guilt, Jealousy, Shame, and Pride), and Neutral. Additionally, the dataset is balanced in terms of the number of recordings per emotion and subject, with the exception of the neutral class, which was not represented at different intensity levels.

In the dataset version used in this work, five subjects were not available (JP_01, JP_02, JP_03, JP_04, and JP_05). As a result, the final subset considered in our experiments contains 92 performers and 10, 211 recordings.

**Figure 6:**
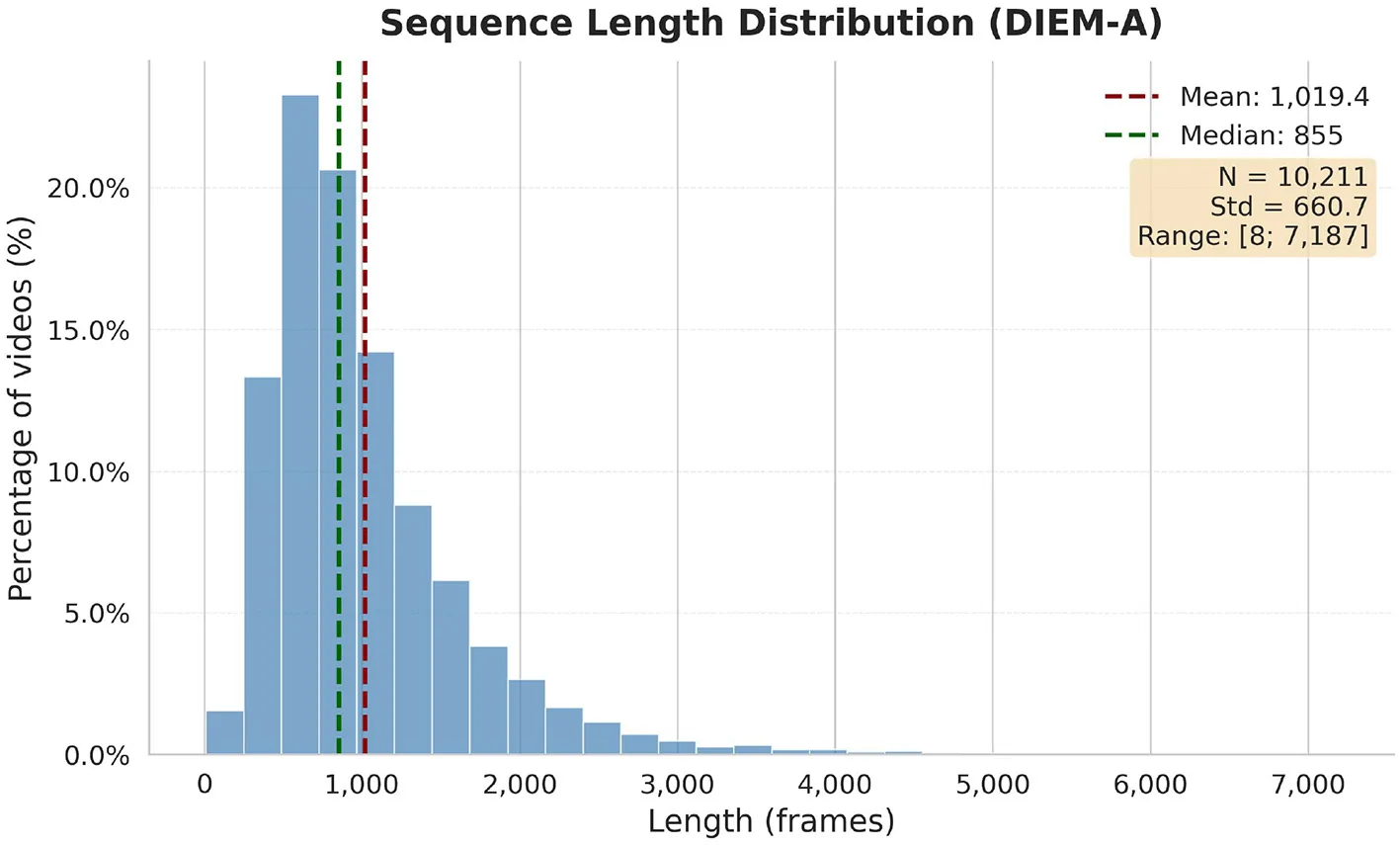
Histogram of the sequence length in the recordings in the DIEM-A dataset.

The performances of the actors are recorded for a duration of 3 to 10 seconds using motion-capture sensors (57 points with x, y, and z cartesian coordinates) although if a performance exceeded the maximum limit, it was still accepted following the “no constraints” principle. As a consequence, the video lengths differ significantly across videos, as shown in Figure 6, where the longest sequence lasts for 7, 187 samples, with an average number of μ = 1, 019.38 samples and a standard deviation of *s* = 660.68 samples.

### 4.2 Experimental conditions

The experiments were conducted under identical experimental conditions. The Graphical Processing Units (GPUs) utilized are a NVIDIA A100 40GB and a NVIDIA L40S 48GB. For the MEED dataset, the results were evaluated following a Leave One Subject Out Cross Validation (LOSO-CV) scheme for 21 subjects and a total of *n* = 1, 326 videos. For the DIEM-A dataset, the results were evaluated following a K-Subject-Fold Cross Validation (KS-Fold-CV) scheme, where a number of subjects are grouped into folds due to the large number of subjects available. In our experiments, we selected *K* = 10 folds to group the 92 subjects for a total of 10, 211 recordings.

### 4.3 Metrics

Although class imbalance is not a major concern in the evaluated datasets, we report Macro-F1 (M-F1) and Weighted-F1 (W-F1) in addition to accuracy, which remains the most commonly reported metric in prior emotion recognition literature. The inclusion of these two F1-based measures provides a more complete view of performance since accuracy alone may obscure differences in how well the model handles each emotion category. In particular, M-F1 assigns equal importance to all classes and is therefore useful for assessing whether performance is consistent across emotions, whereas W-F1 reflects the contribution of each class according to its relative support and provides a complementary global summary.

The per-class F1 score is defined as:


$$\begin{array}{l} \text{F}1=\frac{2\text{TP}}{2\text{TP}+\text{FP}+\text{FN}} \end{array}$$ (6)

where TP, FP, and FN denote the numbers of true positives, false positives, and false negatives, respectively. M-F1 is obtained by averaging the per-class F1 scores, while W-F1 weights each class contribution according to its relative support. Formally, these metrics are defined as:


$$\begin{array}{l} \text{W}\text{-}\text{F}1=\overset{N}{\underset{i=1}{\sum }}w_{i}\text{F}1_{i},\text{ M}\text{-F}1=\frac{1}{N}\overset{N}{\underset{i=1}{\sum }}\text{F}1_{i} \end{array}$$ (7)

where *N* denotes the number of emotion classes, F1_*i*_ is the F1 score of class *i*, and *w*_*i*_ represents the proportion of test samples belonging to class *i*.

To provide an uncertainty estimate and assess whether observed differences are statistically meaningful, we report 95% confidence intervals computed using a normal approximation:


$$\begin{array}{l} p\pm 1.96\sqrt{\frac{p\left(1-p\right)}{n}} \end{array}$$ (8)

where *p* denotes the reported metric value, treated as a proportion in [0, 1], and *n* is the size of the test set. In our experiments, *n* = 1, 326 for MEED and *n* = 10, 211 for DIEM-A.

### 4.4 Results for MEED dataset

We report in Table 2 the performance obtained for different batch size values, up to the point where a drop in performance is observed. This exploration also serves as a direct ablation study to isolate the contribution of our proposed Multiple Instance Learning (MIL) strategy. The standard Transformer baseline relies on uniform mean pooling over the sequence (no MIL), whereas the proposed Window Transformer incorporates the Gated Attention MIL head for dynamic temporal saliency scoring.

**Table 2:** Ablation analysis on MEED for different batch-size configurations with 95% confidence intervals (*n* = 1, 326 videos), comparing the standard Transformer (Baseline/No MIL) against the proposed Window Transformer (With MIL).

Model	Batch size	Acc. (%)	Macro-F1 (%)	Weighted-F1 (%)
Transformer	16	43.04 ± 2.67	42.71 ± 2.66	42.12 ± 2.66
	32	47.68 ± 2.69	47.44 ± 2.69	46.86 ± 2.69
	64	48.71 ± 2.69	47.76 ± 2.69	47.11 ± 2.69
	128	47.21 ± 2.69	46.62 ± 2.69	45.76 ± 2.68
	256	46.87 ± 2.69	45.49 ± 2.68	44.85 ± 2.68
Window transformer	16	53.32 ± 2.68	52.88 ± 2.69	52.17 ± 2.69
	32	54.47 ± 2.68	54.29 ± 2.68	53.62 ± 2.69
	64	54.34 ± 2.68	54.02 ± 2.68	53.39 ± 2.69
	128	52.45 ± 2.69	51.93 ± 2.69	51.10 ± 2.69
	256	48.02 ± 2.69	46.79 ± 2.69	46.04 ± 2.68

The results for Window Transformer are obtained under the same configuration (Window Size = 16, Overlap = 8).

For the baseline Transformer architecture, the best result is achieved with a batch size of 64 reaching an accuracy of 48.71 ± 2.69%. In contrast, when evaluating the contribution of the MIL framework within the Window Transformer, the highest accuracy is obtained with a batch size of 32, reaching 54.47 ± 2.68%. Across all matching batch-size sweeps, the Window Transformer consistently outperforms the non-MIL baseline. This performance difference is statistically significant according to the reported confidence intervals, explicitly demonstrating that learning to weigh non-uniform emotional saliency via MIL provides a critical advantage over standard temporal aggregation.

Furthermore, in Table 3 we analyze different window length configurations for MEED dataset. Building on the previous analysis of batch size configurations, these results further show how the internal temporal segmentation of the Window Transformer affects performance when the batch size is fixed to 32. The best result is obtained with a window size of 24 and an overlap of 12, reaching an accuracy of 56.35 ± 2.67%. Reducing or increasing the window size maintaining a 50% overlap leads to a slight drop in accuracy, remaining statistically comparable but still below the best-performing configuration. The lowest result is observed for the smallest temporal segments, with window size 8 and overlap 4, suggesting that overly short windows may not provide sufficient contextual information for emotion recognition. Overall, these observations are consistent with our previous findings, indicating that the proposed architecture benefits from processing local temporal windows with enough context to capture meaningful motion patterns, while both shorter and longer segmentations appear to be less effective.

**Table 3:** Effect of window size and overlap on Window Transformer performance on MEED using a fixed batch size of 32, with 95% confidence intervals (*n* = 1, 326).

Window size	Overlap	Acc. (%)	Macro-F1 (%)	Weighted-F1 (%)
8	4	52.49 ± 2.69	52.68 ± 2.69	51.87 ± 2.69
12	6	53.55 ± 2.68	53.79 ± 2.68	52.66 ± 2.69
16	8	54.47 ± 2.68	54.29 ± 2.68	53.62 ± 2.69
24	12	56.35 ± 2.67	56.02 ± 2.67	55.36 ± 2.68
32	16	54.29 ± 2.68	53.72 ± 2.68	53.31 ± 2.69

**Figure 7:**
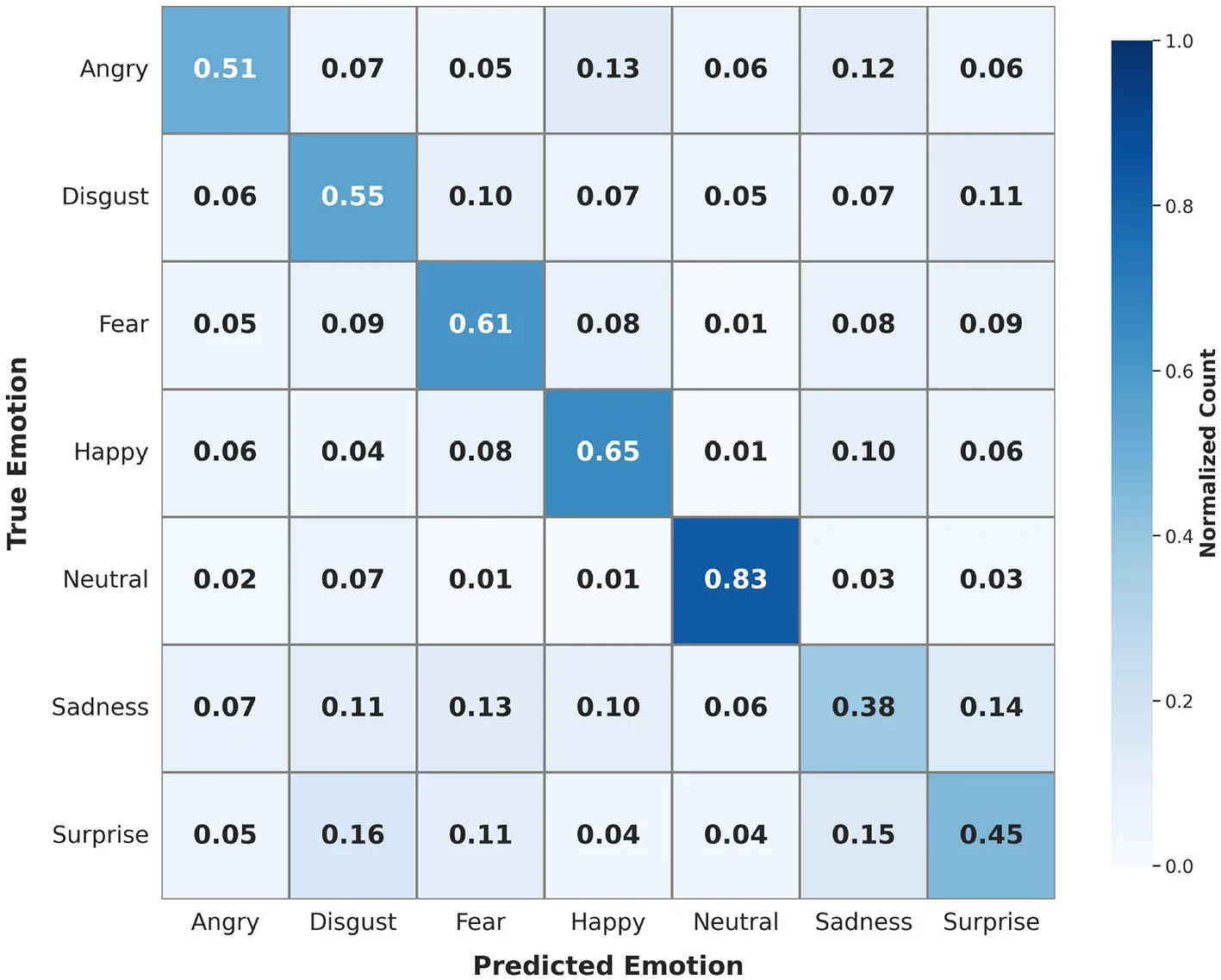
Confusion matrix obtained on the MEED dataset for the best performing Window Transformer configuration (Batch Size = 32 | Window Size = 24), illustrating the distribution of correct and incorrect predictions across emotion classes.

To further analyze the performance of the system, the normalized confusion matrix of the LOSO-CV predictions for the best performing model is provided in Figure 7, showing strong classification in most cases due to the predominant values along the diagonal. However, “Surprise,” “Sadness,” and “Anger” remain the most challenging emotions to identify. In the case of “Anger,” the main sources of confusion occur with “Happy” and “Sadness,” which might be explained by the expressiveness involved in the former and the negativity associated with the latter. For “Surprise,” the model fails to capture it accurately since the predictions are more widely distributed across the emotions with are often conveyed with sudden movements like “Disgust” or “Fear.”

#### 4.4.1 Saliency scorer interpretability

**Figure 8:**
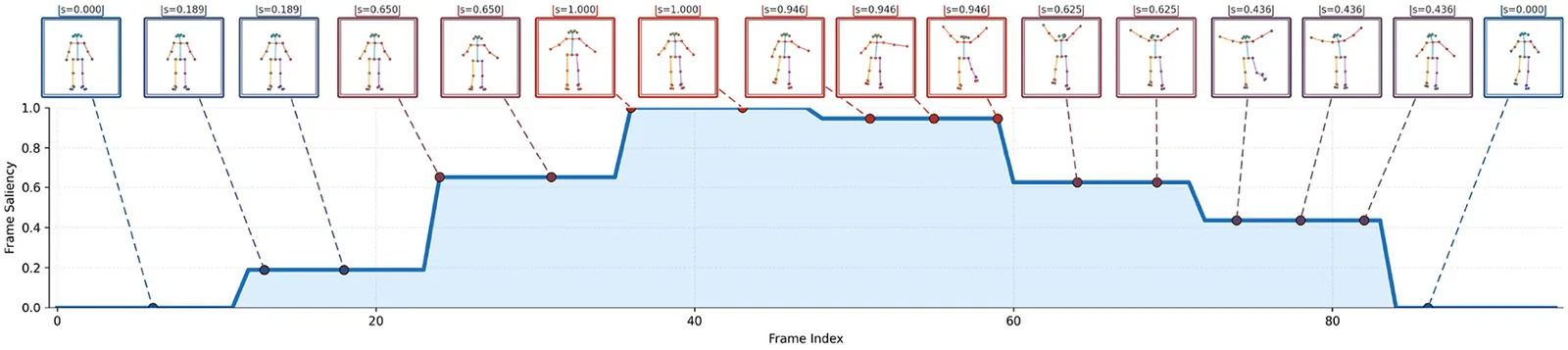
Example of the normalized emotional saliency per window of the video F01H0V3 (Actor ID = F01; Emotion = Happy) taken from MEED dataset using best performing configuration (Batch Size = 32 | Window Size = 24).

Beyond the improvement in performance achieved by the Window Transformer architecture, the saliency values assigned to each window also provide an indication of their contribution to the final classification. Figure 8 illustrates the normalized emotional saliency score associated with each of the windows processed by the model in example from MEED dataset. In particular, greater frame resolution is shown for those segments with higher saliency values in order to better visualize the actor's motion. From this qualitative analysis, the model appears not only to focus on regions with noticeable movement but also to assign higher relevance to those segments that seem to convey stronger emotional content.

### 4.5 Results for DIEM-A dataset

We report in Table 4 the results obtained for different batch size values, up to the point where a drop in performance is observed. As with the previous dataset, this evaluation serves as an ablation study verifying the utility of the Multiple Instance Learning (MIL) mechanism on a structurally distinct sequence corpus.

**Table 4:** Ablation analysis on DIEM-A for different batch-size configurations with 95% confidence intervals (*n* = 10, 211 recordings), comparing the standard Transformer (Baseline/No MIL) against the proposed Window Transformer (With MIL).

Model	Batch size	Acc. (%)	Macro-F1 (%)	Weighted-F1 (%)
Transformer	16	14.18 ± 0.68	12.14 ± 0.63	12.08 ± 0.63
	32	19.02 ± 0.76	18.12 ± 0.75	18.09 ± 0.75
	64	14.77 ± 0.69	11.65 ± 0.62	11.56 ± 0.62
	128	16.07 ± 0.71	13.19 ± 0.66	13.16 ± 0.66
	256^*^	15.89 ± 0.71	13.26 ± 0.66	12.98 ± 0.65
	512^*^	17.54 ± 0.74	15.41 ± 0.70	15.15 ± 0.70
Window transformer	16	23.18 ± 0.82	22.74 ± 0.81	22.81 ± 0.81
	32	22.56 ± 0.81	22.06 ± 0.80	22.19 ± 0.81
	64	22.89 ± 0.81	22.50 ± 0.81	22.58 ± 0.81
	128	22.79 ± 0.81	22.19 ± 0.81	22.34 ± 0.81
	256^*^	23.05 ± 0.82	22.12 ± 0.81	22.35 ± 0.81
	512^*^	22.88 ± 0.81	21.81 ± 0.80	22.07 ± 0.80

The results for Window Transformer are obtained under the same configuration (Window Size = 32, Overlap = 16). The symbol ^*^ indicates that gradient accumulation was applied.

For the standard baseline Transformer architecture (relying on global uniform mean pooling), the best result is achieved with a batch size of 32 obtaining an accuracy of 19.02 ± 0.76%. In contrast, the Window Transformer paired with the Gated Attention MIL head obtains its highest accuracy for a batch size of 16, reaching an accuracy of 23.18 ± 0.82%. Across the entire parameter sweep, the MIL-driven architecture consistently maintains a superior performance profile over the non-MIL baseline. This clear margin demonstrates statistical significance when comparing the highest metrics reported, verifying that learning localized temporal weights remains robust even when changing sequence-processing dimensions.

Building on the Window Transformer architecture, we explore different window size configurations for a fixed batch size of 16 and a 50% fixed overlap across consecutive windows (see Table 5). The evaluated settings show statistically comparable performance, which suggests that the model remains robust across a relatively broad range of temporal segmentations. In particular, these results indicate that a window size of 32 already provides sufficient contextual information for effective emotion recognition, while larger window sizes maintain a similar level of performance without introducing clear gains in accuracy. The highest numerical accuracy is achieved with a window size of 128 and an overlap of 64, reaching 23.84 ± 0.83%.

**Table 5:** Effect of window size and overlap on Window Transformer performance on DIEM-A with batch size fixed to 16, with 95% confidence intervals (*n* = 10, 211).

Window size	Overlap	Acc. (%)	Macro-F1 (%)	Weighted-F1 (%)
32	16	23.18 ± 0.82	22.74 ± 0.81	22.81 ± 0.81
64	32	23.49 ± 0.82	22.98 ± 0.82	23.26 ± 0.82
128	64	23.84 ± 0.83	23.35 ± 0.82	23.48 ± 0.82
256	128	23.69 ± 0.82	23.48 ± 0.82	23.69 ± 0.82
512	256	23.42 ± 0.82	23.29 ± 0.82	23.12 ± 0.82
1,024	512	21.12 ± 0.79	21.00 ± 0.79	21.04 ± 0.79

**Figure 9:**
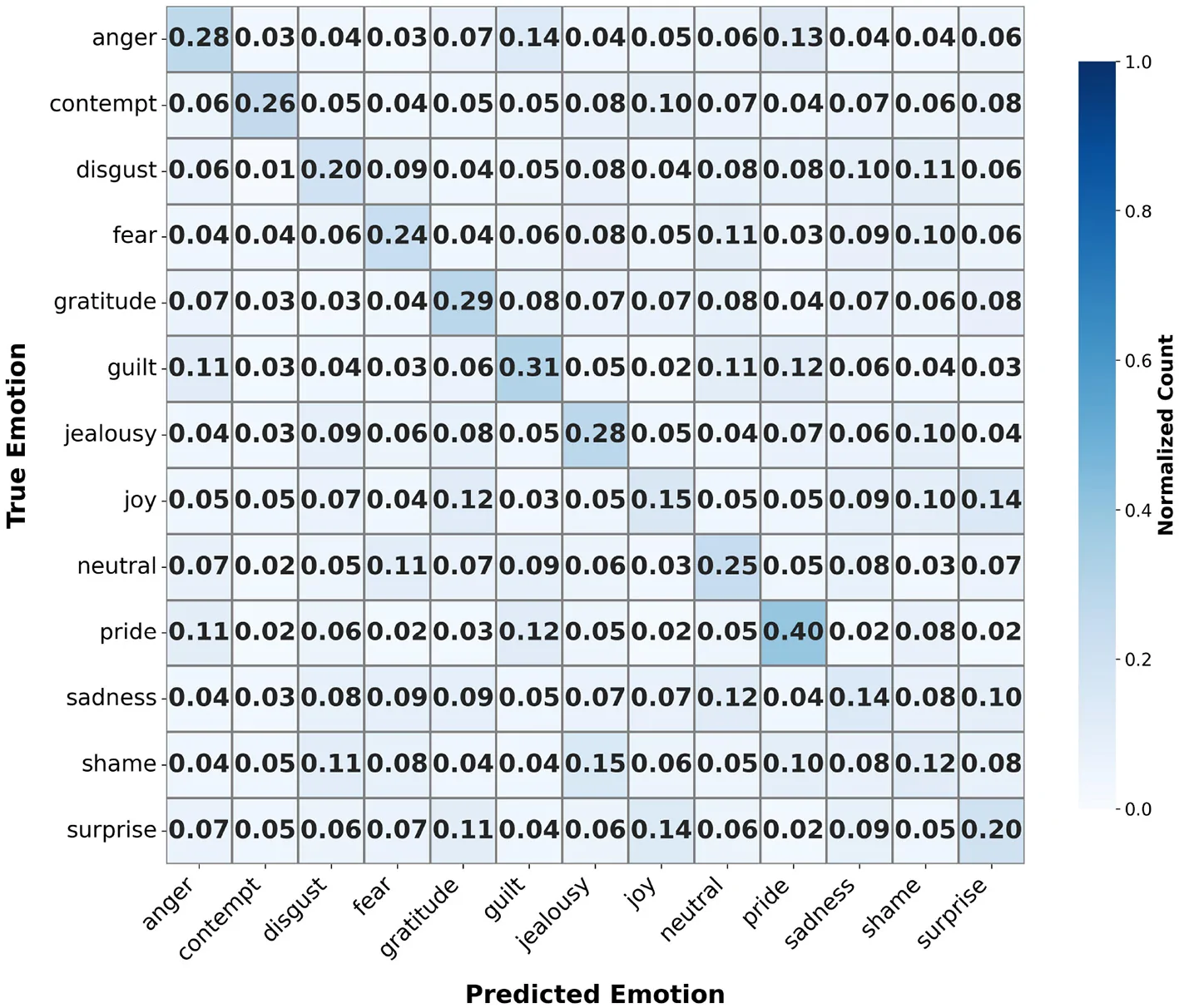
Confusion matrix obtained on the DIEM-A dataset for the best-performing Window Transformer configuration (Batch Size = 16 | Window Size = 128), showing the distribution of predicted labels across the evaluated emotion classes.

To further analyze the performance of the system, the normalized confusion matrix of the KS-Fold-CV predictions for the best-performing model is provided in Figure 9, showing a more challenging classification scenario due to the weaker concentration of values along the diagonal. In this case, “pride” and “guilt” are the emotions that are most accurately identified, whereas classes such as “sadness,” “shame,” and “joy” remain more difficult to recognize. The main sources of confusion tend to occur between emotions with similar affective or expressive characteristics, such as “joy,” “gratitude,” and “surprise,” or among “shame,” “disgust,” and “jealousy.” Overall, the predictions appear more distributed across classes than in MEED, which suggests that DIEM-A poses a more complex recognition problem from body motion alone.

#### 4.5.1 Saliency scorer interpretability

**Figure 10:**
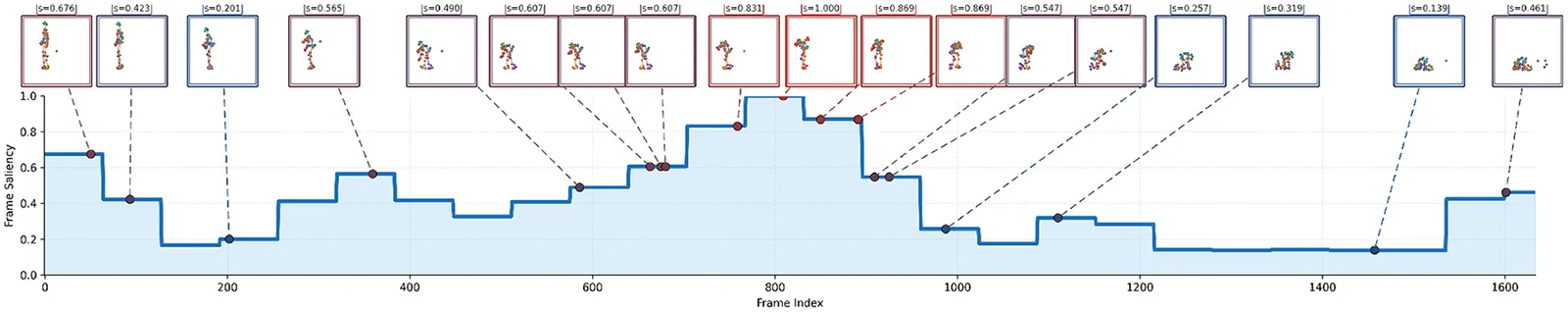
Example of the normalized emotional saliency per window of the video JP_51_fear_2_H (Actor ID = JP_51; Emotion = Fear) taken from the DIEM-A dataset.

In Figure 10, the saliency values assigned by the emotional saliency module are represented together with thumbnails from a motion sequence labeled as “Fear.” From this qualitative analysis, higher saliency values are observed when the performer bends toward the ground, suggesting that the model assigns greater relevance to segments with more expressive body movement for this specific emotion. Nevertheless, it is also noticeable that high saliency values appear toward the end of the sequence, where the performer shows little apparent movement. In this region, some keypoints seem to be incorrectly detected, which may introduce artificial displacements in the motion representation.

These detection errors can be reflected as sudden variations in the velocity and acceleration descriptors, potentially leading the saliency module to assign high relevance to segments that do not necessarily contain meaningful emotional information. This observation highlights one of the limitations of relying on derivative-based motion descriptors when the input landmarks contain noise or missing detections. At the same time, it suggests that increasing the capacity of the modules within the Window Transformer, such as the Transformer encoder and the Saliency Scorer, could improve robustness against noisy keypoints. Alternatively, applying a prior filtering or smoothing stage to remove unreliable landmarks or abrupt artifact-driven motions may also help mitigate this issue without necessarily increasing the number of model parameters.

### 4.6 Ablation study: effectiveness of the MIL strategy

To directly address the contribution of the proposed Multiple Instance Learning strategy, Table 6 provides a consolidated side-by-side comparison isolating the core technical mechanism of this work. We isolate the impact of the dynamic gating MIL strategy by putting the optimized standard Transformer baseline (which uniformly treats the temporal sequence via mean pooling) against the best-performing Window Transformer configuration utilizing the Gated Attention MIL pooling architecture.

**Table 6:** Ablation summary isolating the effect of the proposed Multiple Instance Learning framework.

Dataset	Model configuration	Acc. (%)	Macro-F1 (%)	Weighted-F1 (%)
**MEED**	Baseline Transformer (No MIL)	48.71 ± 2.69	47.76 ± 2.69	47.11 ± 2.69
	**Window transformer (With MIL)**	**56.35** **±2.67**	**56.02** **±2.67**	**55.36** **±2.68**
**DIEM-A**	Baseline Transformer (No MIL)	19.02 ± 0.76	18.12 ± 0.75	18.09 ± 0.75
	**Window transformer (With MIL)**	**23.84** **±0.83**	**23.35** **±0.82**	**23.48** **±0.82**

The table isolates the top configurations for the Baseline Transformer (Uniform Mean Pooling) against the Window Transformer (Gated Attention MIL) across both target datasets with 95% confidence intervals. The best results are highlighted in bold.

As summarized across both independent benchmarks, replacing standard global aggregation with local windowing and MIL-driven saliency weights leads to distinct performance steps. On the MEED dataset, accuracy advances from 48.71 ± 2.69% to 56.35 ± 2.67%, representing an absolute increase of 7.64%. A similarly compelling trend is visible on DIEM-A, where accuracy climbs from 19.02 ± 0.76% to 23.84 ± 0.82%. Crucially, because the underlying frame encoding pipelines remain identical between configurations, these non-overlapping confidence intervals explicitly validate that learning to weight temporally localized emotional markers is a highly effective design decision for multi-instance affective data processing.

### 4.7 Comparison with prior work

For MEED, to the best of our knowledge, no directly comparable recognition results have been reported in the original publication or in subsequent works.

For DIEM-A, direct comparison with previously reported results is limited by differences in dataset configurations, class definitions, and evaluation protocols. The results reported by Cheng et al. (2025) were obtained using human evaluators on a reduced subset of only six subjects from Taiwan, considering the eight basic emotion classes (Joy, Anger, Sadness, Fear, Contempt, Disgust, Surprise, and Neutral) and evaluating only mid-level intensity performances. Additionally, their experiment employed two different point configurations, namely 57 body markers and 18 body points, resulting in a total of 288 videos. Liu et al. (2025) provide an additional DIEM-A reference by evaluating emotion recognition using a LSTM classifier, achieving 34.28% accuracy. However, their work is mainly focused on emotion-conditioned motion generation and uses a specific subset of 49 performers from Japan under an 80/10/10 split. Therefore, the numerical comparisons reported in Table 7 should be interpreted with caution.

**Table 7:** Contextual summary of emotion recognition results on MEED and DIEM-A.

Dataset	Study (paper)	Subset/setting	Classes	Protocol	Acc. (%)
MEED	**Ours**	Full dataset	7	LOSO-CV	**56.35** ± 2.67
DIEM-A	Base (Cheng et al., 2025)	6 Subjects	8	Human evaluators	43.33 ± 5.72
	Liu et al. (2025)	49 Subjects	13	80/10/10 split	34.28 ± 3.99
	**Ours**	6 Subjects	8	KS-Fold-CV	**34.72** ±**7.78**
	**Ours**	92 Subjects	8	KS-Fold-CV	**33.31** ±**1.19**
	**Ours**	92 Subjects	13	KS-Fold-CV	**23.84** ±**0.83**

Only accuracy is reported as the evaluation metric. Values are reported with the corresponding 95% confidence interval margin. Due to differences in subject subsets, class definitions and evaluation protocols, results should be interpreted as dataset-specific references rather than direct comparisons. Our results are highlighted in bold.

Aiming for the closest possible comparison, two additional results are included in Table 7. Firstly, the best-performing Window Transformer model (Batch Size = 16 | Window Size = 128) is trained using only the same 8 emotion classes considered in the results reported by Cheng et al. (2025) with human evaluators, resulting in 6,072 samples. Under this 8-class setting, our model achieves an average emotion recognition accuracy of 33.31 ± 1.19% across all subjects when considering all intensity levels of the selected emotions, where ± denotes the 95% confidence interval margin.

Secondly, six actors from Taiwan are randomly selected from the evaluated subjects, and only their mid-level intensity performances are considered. Following the setting used in the original DIEM-A evaluation, the sampled users are three male (TW_40, TW_30, and TW_08) and three female (TW_15, TW_28, and TW_19) actors. The average emotion recognition accuracy obtained is 34.72 ± 7.78%, where ± denotes the 95% confidence interval margin. This margin is larger than the one reported for Cheng et al. (2025), since our experiment only uses the 57-marker configuration, leading to 144 evaluated samples. Nevertheless, the obtained results provide a more controlled contextual comparison with the human-evaluator setting while still showing that direct numerical comparison remains limited by differences in evaluation conditions.

## 5 Conclusions

In this paper, we propose a Window Transformer architecture grounded in the Multiple Instance Learning (MIL) paradigm for bodily emotion recognition from motion sequences. This formulation represents the sequence as a set of temporal windows, enabling the model to estimate their relevance and assign greater importance to those with higher emotional content, while providing both improved interpretability and more nuanced, context-aware temporal representations for each sequence. This interpretability makes it possible to examine not only how expressive body movements are related to the final emotion label, but also where the most relevant emotional cues appear within the sequence.

Finally, by reporting benchmark results on MEED and DIEM-A datasets, including best accuracies of 56.35 ± 2.67% and 23.84 ± 0.83%, respectively, this work provides reference values that future approaches can build upon, helping to strengthen experimental comparability in bodily emotion recognition. The obtained results highlight the complexity of bodily emotion recognition, especially considering that the models rely exclusively on landmark trajectories and their temporal derivatives, without access to RGB appearance, facial detail, audio or contextual information, which we believe is important for privacy-sensitive applications. This challenge is particularly evident in DIEM-A, where the classification task involves discriminating among 13 different emotions.

Future work may explore alternative temporal aggregation strategies and lightweight mechanisms to improve robustness against noisy or missing landmarks before computing velocity and acceleration descriptors.

## Data Availability

The original contributions presented in the study are included in the article/supplementary material, further inquiries can be directed to the corresponding author.
